# A cathelicidin-related antimicrobial peptide suppresses cardiac hypertrophy induced by pressure overload by regulating IGFR1/PI3K/AKT and TLR9/AMPKα

**DOI:** 10.1038/s41419-020-2296-4

**Published:** 2020-02-06

**Authors:** Xiaofang Wang, Linlin Chen, Xiaoyan Zhao, Lili Xiao, Shanting Yi, Yawei Kong, Yan Jiang, Jinying Zhang

**Affiliations:** 1 Department of Cardiology, the First Afliated Hospital of Zhengzhou University, Zhengzhou, China; 2 Department of Neurology, the First Afliated Hospital of Zhengzhou University, Zhengzhou, China

**Keywords:** Growth factor signalling, Heart failure

## Abstract

Cathelicidin-related antimicrobial peptide (CRAMP), an antimicrobial peptide, was reported to protect against myocardial ischemia/reperfusion injury. However, the effect of CRAMP on pressure overload-induced cardiac hypertrophy was unknown. This study explored the role of CRAMP on cardiac hypertrophy. A cardiac hypertrophy mouse model was induced by aortic banding surgery. Seven days after surgery, mice were given mCRAMP by intraperitoneal injection (8 mg/kg/d) for 7 weeks. Cardiac hypertrophy was evaluated by the hypertrophic response and fibrosis level as well as cardiac function. Mice were also injected with AAV9-shCRAMP to knockdown CRAMP in the mouse heart. CRAMP levels first increased and then reduced in the remodeling heart, as well as in angiotensin II-stimulated endothelial cells but not in cardiomyocytes and fibroblasts. mCRAMP protected against the pressure overload-induced cardiac remodeling process, while CRAMP knockdown accelerated this process. mCRAMP reduced the inflammatory response and oxidative stress in the hypertrophic heart, while mCRAMP deficiency deteriorated the pressure overload-induced inflammatory response and oxidative stress. mCRAMP inhibited the angiotensin II-stimulated hypertrophic response and oxidative stress in neonatal rat cardiomyocytes, but mCRAMP did not help the angiotensin II-induced inflammatory response and oxidative stress in endothelial cells. Mechanistically, we found that mCRAMP suppressed the cardiac hypertrophic response by activating the IGFR1/PI3K/AKT pathway via directly binding to IGFR1. AKT knockout mice completely reversed the anti-hypertrophic effect of mCRAMP but not its anti-oxidative effect. We also found that mCRAMP ameliorated cardiac oxidative stress by activating the TLR9/AMPKa pathway. This was confirmed by a TLR9 knockout mouse experiment, in which a TLR9 knockout partly reversed the anti-hypertrophic effect of mCRAMP and completely counteracted the anti-oxidative effect of mCRAMP. In summary, mCRAMP protected against pressure overload-induced cardiac hypertrophy by activating both the IGFR1/PI3K/AKT and TLR9/AMPKa pathways in cardiomyocytes.

## Introduction

Cardiac hypertrophy involves alterations in shape, size, and function of the heart after injury or stress, such as myocardial infarction, myocardial ischemia, hypertension, myocarditis, and cardiomyopathy^1^. It is the pathological process of heart failure (HF), which initially is beneficial for cardiac function, but later leads to maladaptation^2^. Physiological hypertrophy occurs in healthy people after exercise or pregnancy and is classified as “physiological hypertrophy” because the heart is not damaged^3^. However, when a heart insult occurs, the heart starts the pathological hypertrophy process, which involves alterations in fetal gene expression, changes in expression levels of protein energetic and metabolic states, as well as fibrosis and cardiac dysfunction^4^. For decades, clinical drugs including angiotensin converting enzyme inhibitor, angiotensin receptor blockade, and beta-receptor blockade have been used for HF; however, the morbidity and mortality of HF is gradually increasing^5^. Thus, finding new therapeutic targets for HF is of great importance.

It has long been appreciated that physiological hypertrophy imposed by disease is distinctly different from pathological hypertrophy, which does not cause myocardial fibrosis or changes in embryonic and adult gene expression, nor cardiac dysfunction^6^. It is possible that the signaling pathway involved in physiological hypertrophy protects the heart from worse insults. The central signals of physiological hypertrophy are insulin-like growth factor-1 (IGF-1) and the growth hormone (GH) mediated phosphoinositide 3 kinase (PI3K)/Akt signaling pathway^7^. Persistent PI3K-activated cardiac hypertrophic transgenic mice exhibit cardiac enlargement, but do not have fibrosis and fetal gene re-expression^8^. Akt1 deficient mice are resistant to exercise-induced cardiac hypertrophy, but they develop greater cardiac hypertrophy under pressure overload^9^. Thus, regulating the central pathway of physiological hypertrophy may be a new method for regulating HF.

The antimicrobial peptide cathelicidin (CRAMP in mouse/rat, LL-37 in human) is expressed in many immune cells, such as epithelial cells, and genital cells^10^. CRAMP was initially reported as an immunomodulatory peptide that functions in many autoimmune diseases, such as psoriasis^11^, systemic lupus erythematous^12^, arthritis^13^, atherosclerosis^14^, and type 1 diabetes^15^. Recent studies have identified a role of CRAMP in cardiovascular disease. Yihua Bei reported that CRAMP could protect against a cardiac ischemia/reperfusion injury^16^. CRAMP participates in the pathogenesis of atherosclerosis. CRAMP was also found to ameliorate cardiac dysfunction in myocardial infarction^17^. However, the role of CRAMP in cardiac hypertrophy was unclear. Thus, we used an aortic banding model to establish a pathological cardiac hypertrophy mouse model to explore the functional role of CRAMP.

## Methods

### Animals and animal models

All animal experiments adhered to the Animal Guide for the Care and Use of Laboratory Animals published by the US National Institutes of Health (NIH Publication No. 85-23, revised 1996) and the Animal Care and Use Committee of Zhengzhou University. Aortic banding (AB) surgery was used to construct a cardiac hypertrophy model as previously described^18^. The Chinese Academy of Medical Sciences (Beijing, China) provided 8–10-week-old male C57BL/6 mice (23.5–27.5 g). mCRAMP was administered by intraperitoneal injection to the animals 7 days after the induction of cardiac hypertrophy (8 mg/kg/d) and until 8 weeks after AB surgery. An equal volume of saline was administered to the control mice. Mice were injected with AAV9-shCRAMP at the same time of AB surgery to knockdown CRAMP. AKT1 knockout (KO) (stock number: 004912) mice were purchased from Jackson Laboratory; and TLR9 knockout mice were purchased from the model animal research center of Nanjing University. AKT1-KO and TLR9-KO mice were also subjected to AB surgery and then administered CRAMP injection (for the same time period, as above). Then, mice were sacrificed and the hearts were removed.

### Adeno-associated virus vector

Recombinant AAV9-shCRAMP was constructed by the Vigene Bioscience Company (Jinan, China). Briefly, the cytomegalovirus (CMV) promoter was used to drive the expression of shCRAMP. A plasmid was constructed to generate AAV9-shCRAMP viral particles via triple transfection of HEK 293T cells. Iodixanol gradient centrifugation and FPLC were used to purify the plasmid. Quantitative real-time PCR was used to determine titers of the AAV vectors (viral genomes/mL). AAV9-LacZ was used as a control, which was packed with an empty AAV9 virus. Mice received 5 × 10^11^ vp, viral particles per animal of AAV9-shCRAMP by myocardial injection, as previous described^19^.

### Echocardiographic analyses

The left ventricular diameter and wall thickness were evaluated using a Mylab 30 cv ultrasound (Esoate S.p.A), with a 10 MHz linear array ultrasound probe, as previously described^18,20^.

### Quantitative real-time RT-PCR and western blot

Total RNA (2 μg per sample) from frozen mouse heart tissue and cardiomyocytes was reverse transcribed into cDNA using the oligonucleotide (DT) primer and the transcript first strand cDNA synthesis kit (Roche). Then, a light Cycler 480 instrument (software version 1.5, Roche) and the SYBR green PCR master mix (Roche) was used to perform RT-PCR. All genes were normalized using GAPDH.

Protein samples (50 μg) from heart tissue and cardiomyocytes were lysed in RIPA lysis buffer, separated by SDS-PAGE, and transferred to a PVDF membrane (Millipore, Beijing, China). Different primary antibodies were added including CRAMP, phosphorylated (P-) and total (T-) IGFR1, PI3K, AKT1, P-AMPKα (purchased from Cell Signaling Technology, 1:1000 dilution), TLR4, TLR7, TLR9, keap1, NRF2, NOX2, NOX4, laminB, and GAPDH (purchased from Abcam, 1:1000 dilution). Chemiluminescence (ECL) reagents (Bio-Rad, Hercules, CA, USA) were used for detection and blots were imaged using a ChemiDoc MP Imaging System (Bio-Rad).

### Histological analysis

The heart was removed and fixed with 10% paraformaldehyde and embedded in paraffin. After sectioning (5-μm thick), hematoxylin and eosin (H&E) staining was used to calculate myocardial cell area, and picric acid red (PSR) staining was used to calculate collagen deposition. Calculations of cardiomyocyte area (at least 200 per group) and collagen deposition rate were performed using a quantitative digital image analysis system (Image Pro-Plus, IPP, version 6.0).

### Cell isolation and culture

Neonatal rat cardiomyocytes (NRCMs) and fibroblasts (CFs) were isolated as previously described^18^. One- to three-day-old Sprague-Dawley rat-derived NRCMs and CFs were seeded (1 × 10^6^ cells/well) into a 6-well plate with DMEM/F12 with 10% fetal bovine serum (FBS). Then, cells were serum-starved for 8 h, followed by treatment with Angiotensin II (Ang II, 1 μΜ, Sigma) for an additional 12, 24, or 48 h. NRCMs were treated with CRAMP (0.05, 0.15, or 0.5 mg/L) for 24 h. NRCMs were transfected with AKT1 siRNA and TLR9 siRNA (Santa Cruz) to knockdown AKT1 and TLR9, respectively. Cell viability was detected by MTT assay (Beyotime, Beijing, China). NRCMs were stained with α-actin to detect cell surface area.

Primary adult mouse heart endothelial cells (MHECs) were isolated as previously described^21^. Briefly, 4–6-week-old mouse hearts were cut in Hanks’ balanced salt solution buffer. Then, hearts were digested in collagenase A and digestion was stopped with 10% FBS-DMEM. MHECs were isolated using CD31 beads. Cells were then washed and cultured in a pre-coated well with 2% gelatin (Sigma, Oakville, ON, Canada). MHECs were treated with mCRAMP (0.05, 0.15, or 0.5 mg/L) and Ang II (1 μΜ) for 24 h.

### Oxidative stress (OS)

The level of reactive oxygen species (ROS) was measured according to a previous study^22^ using 2ʹ,7ʹ-dichlorofluorescin diacetate (DCFH-DA) and an ELISA plate reader (Synergy HT, BioTek, Vermont, USA). Commercial reagents were used to detect total superoxide dismutase (SOD), glutathione peroxidase (Gpx), and NADPH activity. Commercial kits (Beyotime, Beijing, China) were used to detect oxidase and anti-oxidase activity. Dihydroethidium (DHE) staining (Sigma Aldrich) was performed to identify intracellular ROS production. Mean DHE fluorescence was calculated by subtracting integrated density of the background signal from the integrated density of the fluorescent staining from 10 fields/heart, 5 hearts/group and normalized to control.

### Enzyme-linked immunosorbent assay (ELISA)

ELISA kits were used to test the release of TNFα, IL-1, and IL-6 (BioLegend) according to a previous study^22^. The optical density at 450 nm was detected by an ELISA plate reader.

### Co-immunoprecipitation assays

NRCMs were transfected with psicoR-HA-CRAMP and psicoRFlag-IGFR1 or psicoRFlag-TLR9. An immunoprecipitation buffer was used to lyse cells. A/G agarose beads and 1 μg antibody were used to bind protein overnight at 4 °C. Then, other antibodies were used to immunoprecipitate the eluted proteins.

### Statistical analysis

All data are expressed as the mean ± SD. The group sizes of the in vivo experiments were estimated based on power analysis of HW/BW with an α error of 5% and a power of 80%. To detect a 10% change in HW/BW with an expected SD of 5%, we needed five animals per group. In our study, *n* = 10 fulfilled the statistical requirements. A random number table method was used to assign mice. All experiments were blinded to all operators. Comparisons between two groups were conducted using an unpaired Student’s *t* test. Comparisons between groups were conducted by one-way ANOVA. *P* < 0.05 was considered to be statistically significant.

## Results

### Expression levels of CRAMP in a hypertrophic heart

**Fig. 1:**
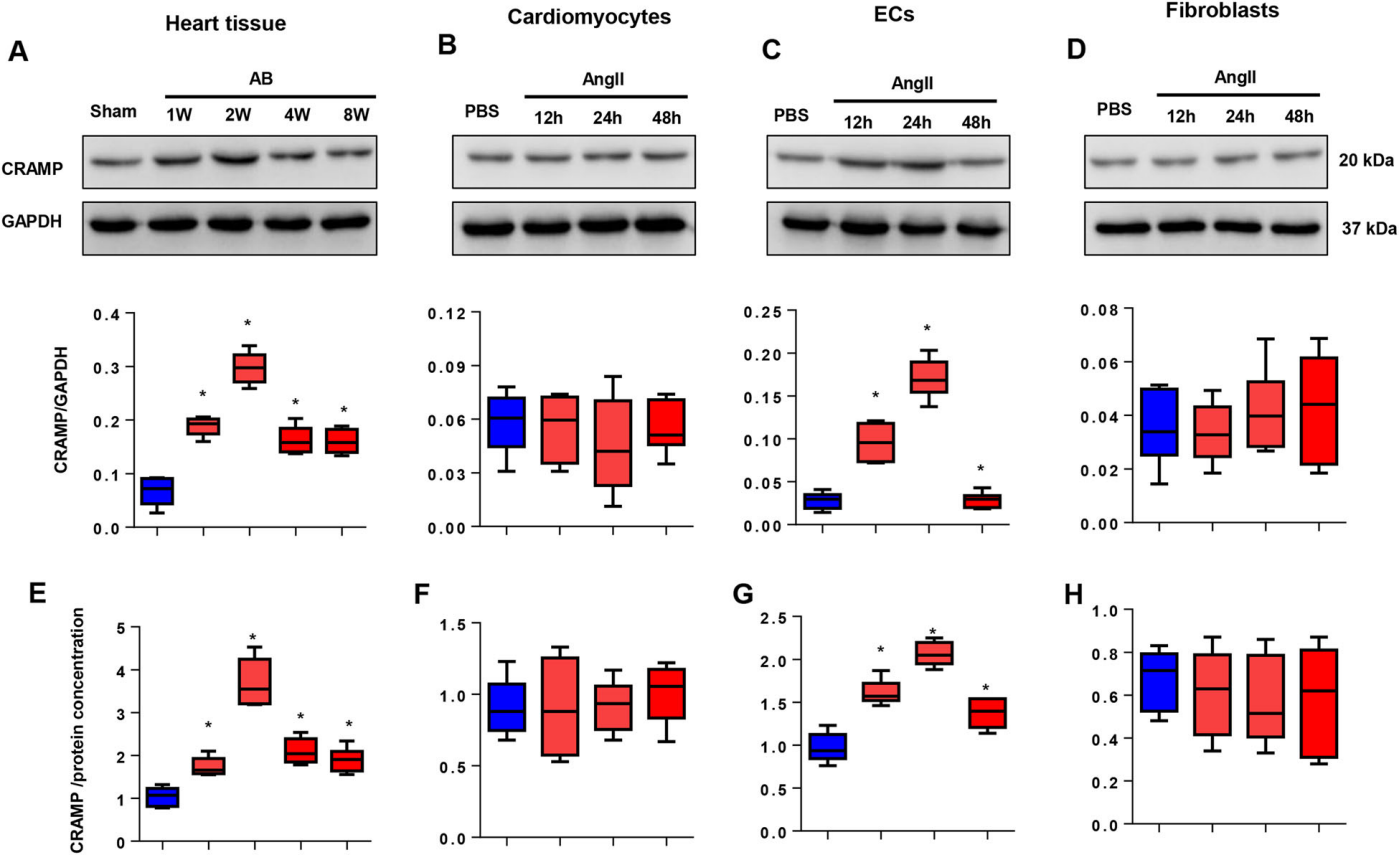
Expression levels of CRAMP in a hypertrophic heart. **a**, **e** Protein levels and concentration of CRAMP in heart tissue undergoing AB (*n* = 6). **b**, **f** Protein levels and concentration of CRAMP in NRCMs treated with Ang II (1 μM) (*n* = 6). **c**, **g** Protein levels and concentration of CRAMP in MHECs treated with Ang II (1 μM) (*n* = 6). **d**, **h** Protein levels and concentration of CRAMP in fibroblasts treated with Ang II (1 μM) (*n* = 6). *P < 0.05 vs. sham/PBS.

To elucidate the functional role of CRAMP on cardiac hypertrophy, we first detected the expression levels of CRAMP in a hypertrophic heart. As shown in Fig. 1a, the expression of CRAMP was sharply increased after 1 week after AB surgery, peaked at 2 weeks after AB surgery, and then decreased at 4 weeks after AB, until 8 weeks after AB surgery. The protein expression of CRAMP at 1, 2, 4, and 8 weeks after AB surgery was relatively higher than the sham group. We then isolated NRCMs, MHECs and CFs and treated these cells with Ang II for 12, 24, and 48 h. As a result, the expression of CRAMP increased in cardiomyocytes after Ang II stimulation, but was not significantly different (Fig. 1b). The same result was observed in CFs, in which we observed an increase of CRAMP protein levels, but it was not statistically significant (Fig. 1d). Interestingly, we found the expression pattern in MHECs was much more consistent with that seen in heart tissue, where CRAMP expression started to increase 12 h after Ang II stimulation to 24 h, and then dropped 48 h after stimulation (Fig. 1c). We also used ELISA assays to detect the CRAMP concentration in heart tissue and the three cell types. Consistent with the western blot results, the expression of CRAMP was sharply increased 1 week after AB surgery, peaked at 2 weeks after AB surgery, and then decreased 4 weeks after AB surgery in a hypertrophic heart (Fig. 1e). CRAMP peptide concentration was increased at 12–24 h after stimulation, and then dropped after 48 h of stimulation in MHECs (Fig. 1g). No significant difference in expression of CRAMP peptide was found in NRCMs and CFs after stimulation (Fig. 1f, h). These data indicated that CRAMP derived from MHECs may participate in the pathological process of cardiac hypertrophy.

### CRAMP treatment ameliorated cardiac hypertrophy

**Fig. 2:**
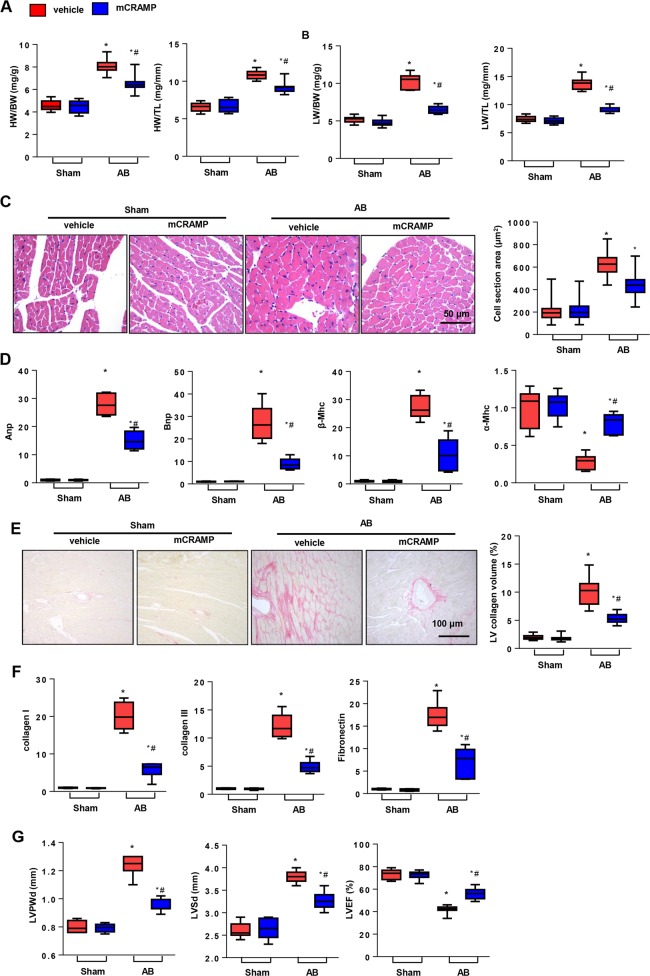
CRAMP treatment ameliorated cardiac hypertrophy. **a** HW/BW, HW/TL in mice undergoing 8 weeks of AB treatment with CRAMP (*n* = 10). **b** LW/BW, LW/TL in mice (*n* = 10). **c** H&E staining (*n* = 6) and cell cross-sectional area (*n* > 200 cells/group) in mice. **d** Fetal gene transcription level (*n* = 6). **e** PSR staining (*n* = 6) and LV collagen volume in mice. **f** Fibrosis gene transcription level (*n* = 6). **g** Echocardiography analysis (*n* = 8). *P < 0.05 vs. vehicle-sham; ^#^*P* *<* 0.05 vs. vehicle-AB.

Thus, we used mCRAMP to treat mice 7 days after AB surgery until 8 weeks after surgery to observe the role of CRAMP on cardiac hypertrophy. As shown in Fig. 2a, the heart weight indicated by heart weight to body weight or total length ratios was reduced in the CRAMP treatment group when compared with the vehicle group 8 weeks after AB. Lung weight as a character of pulmonary edema, indicated by lung weight to body weight or total length ratios was also reduced in the CRAMP treatment group when compared with the vehicle-AB group (Fig. 2b). H&E staining was used to count the cell cross-sectional area. We observed a decrease in the cell cross-sectional area in the CRAMP treatment group when compared with the vehicle-AB group (Fig. 2c). The re-expression of fetal genes were also evaluated by RT-PCR, as observed in Fig. 2d. The re-expression of atrial natriuretic peptide (Anp), B-type natriuretic peptide (Bnp), and myosin heavy chain β (β-Mhc) was decreased in the CRAMP treatment group, while the reprogramming of myosin heavy chain α (α-Mhc) was improved by CRAMP treatment. Cardiac fibrosis is a second feature of cardiac hypertrophy, which leads to wall stiffness. PSR staining was performed to detect cardiac staining. As shown in Fig. 2e, the left ventricular (LV) collagen volume was decreased by CRAMP treatment. The expression of the fibrosis markers collagen I, collagen III, and fibronectin was also decreased by CRAMP treatment (Fig. 2f). Cardiac function was detected 8 weeks after surgery. The LV post wall dimension (LVPWd), and LV systolic diameter (LVSd) were decreased by CRAMP treatment, but the LV ejection fraction (LVEF) was improved by CRAMP treatment (Fig. 2g).

### CRAMP deficiency accelerated cardiac hypertrophy

**Fig. 3:**
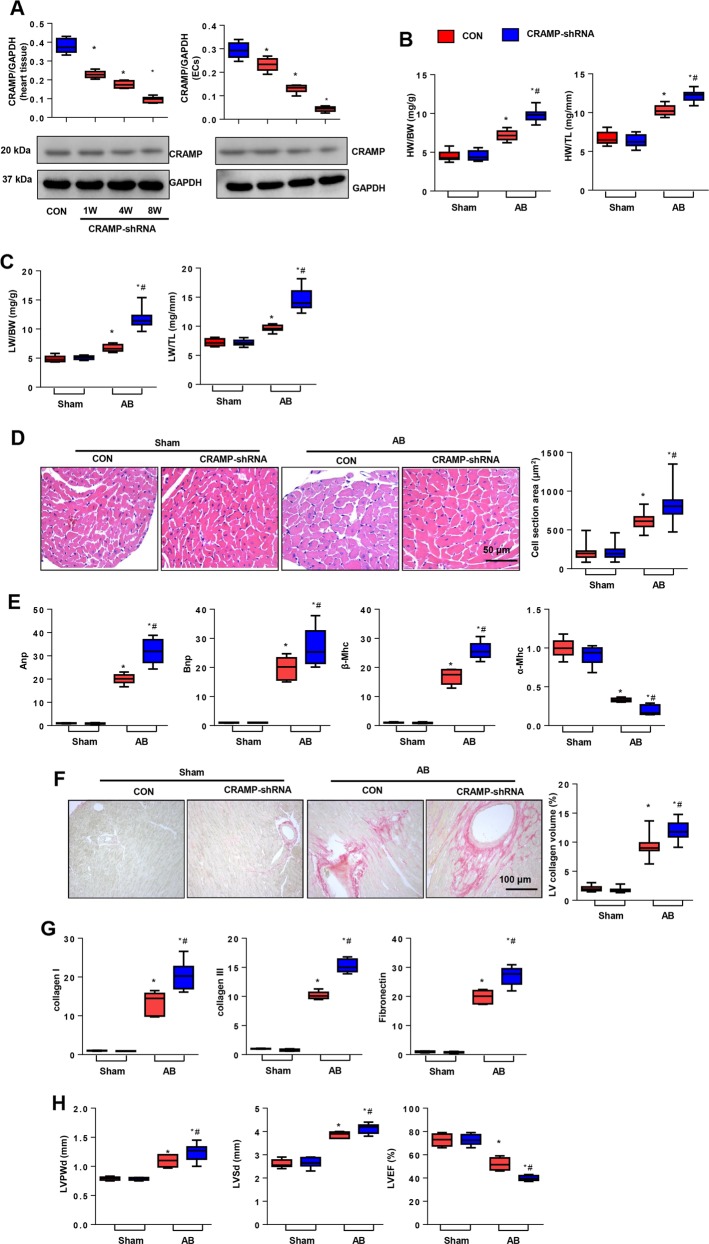
CRAMP deficiency accelerated cardiac hypertrophy. **a** CRAMP levels in mouse heart and MHECs after injection with AAV9-shCRAMP for 1, 4, or 8 weeks. **b** HW/BW, HW/TL in mice undergoing 8 weeks of AB injected with AAV9-shCRAMP (*n* = 10). **c** LW/BW, LW/TL in mice (*n* = 10). **d** H&E staining (*n* = 6) and cell cross-sectional area (*n* > 200 cells/group) in mice. **e** Fetal gene transcription level (*n* = 6). **f** PSR staining (*n* = 6) and LV collagen volume in mice. **g** Fibrosis gene transcription level (*n* = 6). **h** Echocardiography analysis (*n* = 8). *P < 0.05 vs. CON-sham; ^#^*P* < 0.05 vs. CON-AB.

We then validated whether CRAMP deficiency would show the opposite effect. Mice were subjected to myocardial injection of AAV9-shCRAMP and AB surgery at the same time. As shown in Fig. 3a, the expression of CRAMP in heart tissue and MHECs was sharply decreased at 1, 4, and 8 weeks after injection. The heart and lung weights were remarkably increased in the CRAMP deficient group (Fig. 3b, c). The cell cross-sectional area was sharply enlarged in the CRAMP deficient group (Fig. 3d). Re-expression of the fetal genes was augmented and adult genes were reduced in the CRAMP deficient group (Fig. 3e). Fibrosis also deteriorated in the CRAMP deficient group (Fig. 3f, g). LVPWd and LVSd increased, and LVEF was reduced in the CRAMP deficient group (Fig. 3h).

### CRAMP affects inflammation and oxidative stress in the heart

**Fig. 4:**
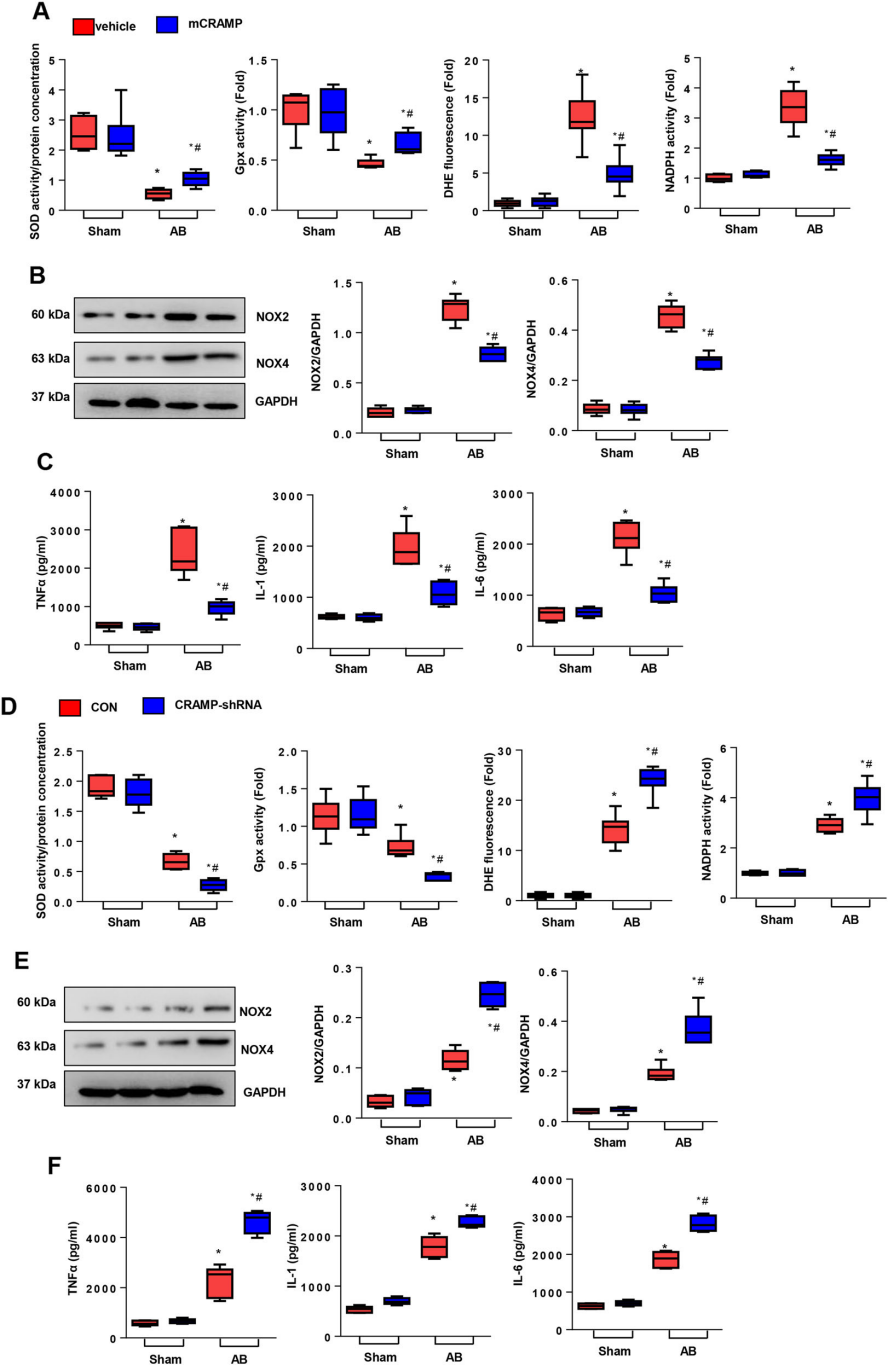
CRAMP affected inflammation and oxidative stress in the heart. **a** SOD, Gpx, DHE and NADPH oxidase activity in mouse heart 8 weeks after AB surgery and treatment with CRAMP (*n* = 6). **b** NOX2 and NOX4 level in heart tissue treated with CRAMP (*n* = 6). **c** TNFα, IL-1, IL-6 levels in mouse heart 8 weeks after AB surgery and treatment with CRAMP (*n* = 6). *P < 0.05 vs. vehicle-sham; ^#^*P* < 0.05 vs. vehicle-AB. **d** SOD, Gpx, DHE, and NADPH oxidase activity in mouse heart 8 weeks after AB surgery and injection with AAV9-shCRAMP (*n* = 6). **e** NOX2 and NOX4 level in heart tissue injected with AAV9-shCRAMP (*n* = 6). **f** TNFα, IL-1, IL-6 levels in mouse heart 8 weeks after AB surgery and injection with AAV9-shCRAMP (*n* = 6). *P < 0.05 vs. CON-sham; ^#^*P* < 0.05 vs. CON-AB.

OS and inflammation are other features of HF. Since CRAMP was first identified as an anti-inflammatory peptide, we detected the association between CRAMP and OS and inflammation in a hypertrophic heart. As shown in Fig. 4a, b, CRAMP treatment increased the anti-oxidase activity (SOD2 and Gpx), and decreased ROS level (DHE), reduced oxidase activity (NADPH) and NOX2, NOX4 level in a hypertrophic heart. CRAMP also decreased pro-inflammatory cytokine release (TNFα, IL-1, and IL-6) in a hypertrophic heart (Fig. 4c). Conversely, CRAMP deficiency deteriorated OS and inflammation in a failing heart (Fig. 4d–f). These results indicate that OS and inflammation also participate in the protective effect of CRAMP on HF.

### Effects of CRAMP on cardiomyocytes and MHECs

**Fig. 5:**
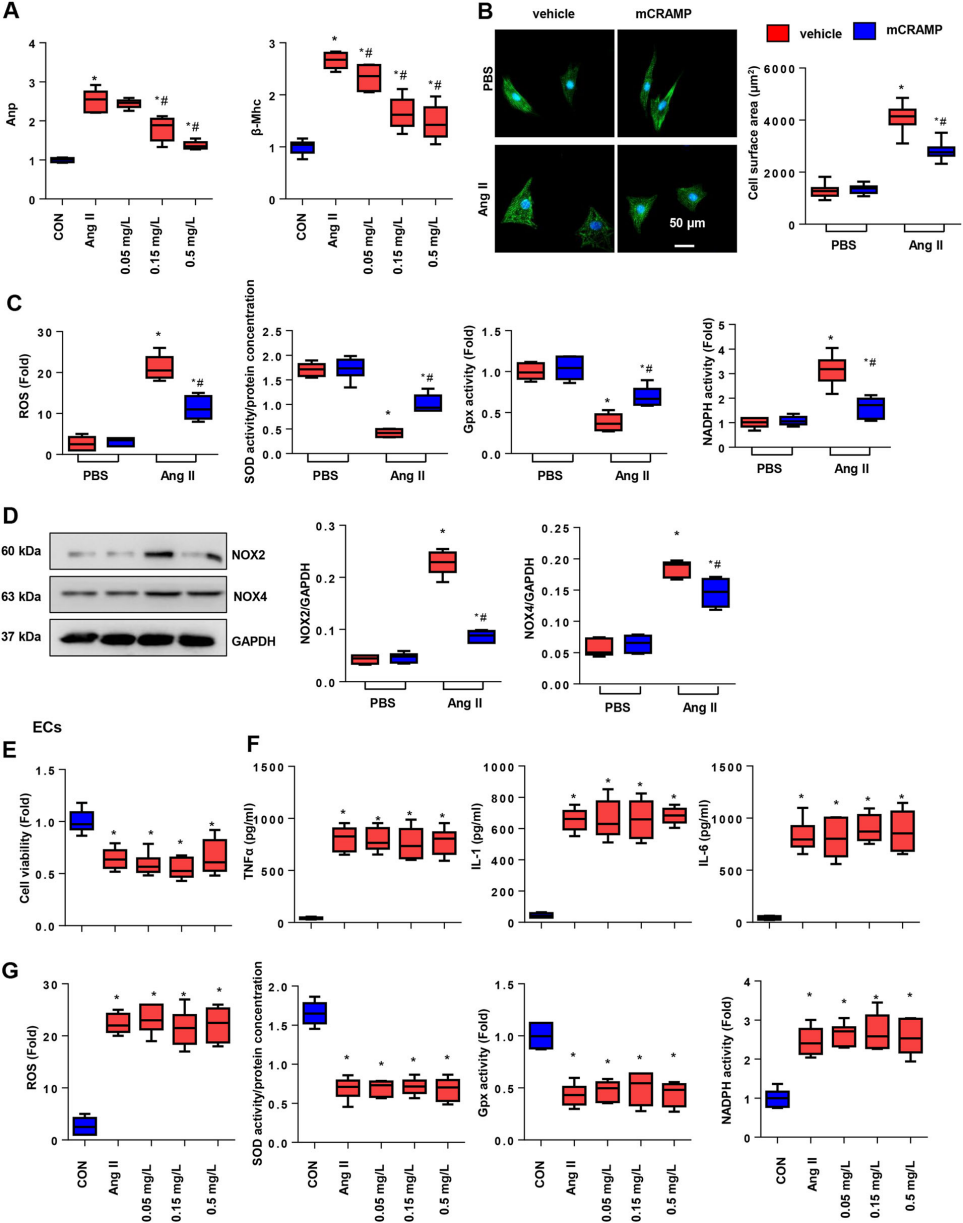
Effects of CRAMP on cardiomyocytes and MHECs. **a** NRCMs were treated with CRAMP (0.05, 0.15, or 0.5 mg/L) for 24 h. Fetal gene transcription (*n* = 6). **b**, **c** NRCMs were treated with CRAMP (0.5 mg/L) and Ang II (1 μM) for 24 h. **b** α-Actin staining (*n* = 6) and cell surface area (*n* > 50 cells per group). **c** ROS levels, SOD, Gpx, and NADPH oxidase activity in NRCMs (*n* = 6). **d** NOX2 and NOX4 level in NRCMs (*n* = 6). **e**–**g** MHECs were treated with CRAMP (0.05, 0.15, or 0.5 mg/L) and/or Ang II (1 μM) for 24 h. **e** Cell viability (*n* = 6). **f** TNFα, IL-1, IL-6 levels in MHECs (*n* = 6). **g** ROS levels, SOD, Gpx, and NADPH oxidase activity in MHECs (*n* = 6). *P < 0.05 vs. CON; ^#^*P* < 0.05 vs. Ang II.

Since the alteration of CRAMP expression on a failing heart was mainly observed in MHECs, we detected the effect of CRAMP on both MHECs and cardiomyocytes. As shown in Fig. 5a, Ang II induced an increased hypertrophic response of NRCMs, while CRAMP treatment (0.05, 0.15, or 0.5 mg/L) revealed a dose-dependent anti-reprogramming of fetal genes. Thus, we chose 0.5 mg/L CRAMP to treat NRCMs. As shown in α-actin staining, the cell surface area was sharply decreased by CRAMP treatment. We also detected OS in CRAMP-treated NRCMs. As shown in Fig. 5c, d, the ROS level was decreased by CRAMP treatment, anti-oxidase activity increased, and oxidase activity decreased with CRAMP treatment in cardiomyocytes. These data indicate that CRAMP derived from MHECs directly target cardiomyocytes. We then isolated MHECs to evaluate the role of CRAMP on MHECs. As shown in Fig. 5e, CRAMP treatment (0.05, 0.15, or 0.5 mg/L) did not affect Ang II-induced low cell viability. We also detected OS and the inflammatory response in MHECs stimulated with Ang II. As a result, CRAMP treatment (0.05, 0.15, or 0.5 mg/L) did not affect the Ang II-induced OS and inflammation response in MHECs (Fig. 5f, g). These data indicate that CRAMP derived from MHECs has no effect on MHECs themselves, but functions on cardiomyocytes.

### CRAMP activated IGFR1/PI3K/AKT1 signaling

**Fig. 6:**
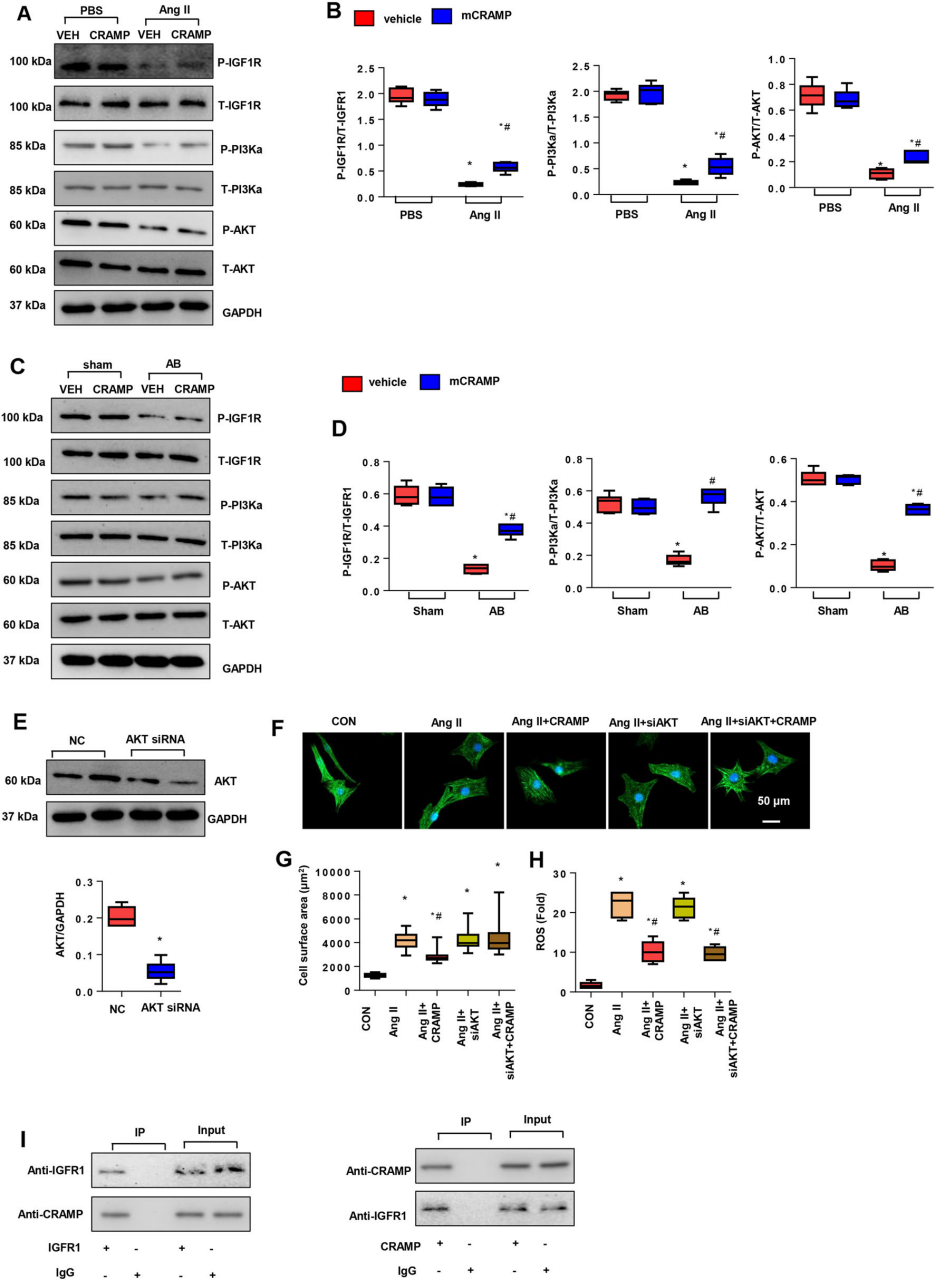
CRAMP activated IGFR1/PI3K/AKT1 signaling. **a**, **b** Protein levels of IGF1R, PI3K, AKT1 in NRCMs treated with mCRAMP (0.5 mg/L) and Ang II (1 μM) (*n* = 6). *P < 0.05 vs. vehicle-CON; ^#^*P* < 0.05 vs. vehicle-Ang II. **c**, **d** Protein levels of IGF1R, PI3K, AKT1 in remodeling heart tissue treated with mCRAMP (*n* = 6, *P < 0.05 vs. CON-sham; ^#^*P* < 0.05 vs. CON-AB). **e**–**i** NRCMs were transfected with AKT1 siRNA and treated with mCRAMP (0.5 mg/L) and Ang II (1 μM). **e** Protein levels of AKT1 in NRCMs (*n* = 6). **f**, **g** α-actin staining (*n* = 6) and cell surface area (*n* > 50 cells per group). **h** ROS levels (*n* = 6). *P < 0.05 vs. CON; ^#^*P* < 0.05 vs. Ang II. **i** Co-IP experiment in NRCMs.

The anti-hypertrophic mechanism of CRAMP was analyzed. Studies have reported CRAMP can bind to IGFR1^23^, thus we detected the central signaling pathway of physiological cardiac hypertrophy. As shown in Fig. 6a–d, decreased phosphorylation of IGFR1, PI3K, and AKT1 was observed in both Ang II-stimulated cardiomyocytes and remodeling heart tissue. CRAMP treatment increased the phosphorylation levels of these proteins. To confirm this is the only way that CRAMP functions in cardiac hypertrophy, we used AKT1 siRNA to silence AKT1 in NRCMs (Fig. 6e). We found the anti-hypertrophic effect of CRAMP was blocked by AKT1 siRNA, but the anti-OS effect of CRAMP was not affected, as shown by increased cell surface area, but decreased ROS levels in AKT1 silenced CRAMP-treated cells (Fig. 6f–h). We performed co-IP experiments to detect whether CRAMP could directly bind IGFR1 in cardiomyocytes. As shown in Fig. 6i, the results showed a positive binding of CRAMP to IGFR1 in NRCMs. These data suggested that the anti-hypertrophic role of CRAMP was mediated by activation of IGFR1/PI3K/AKT1 by directly binding to IGFR1. However, the mechanism of the anti-OS role of CRAMP was still unknown.

### CRAMP affected TLR9/AMPKα

**Fig. 7:**
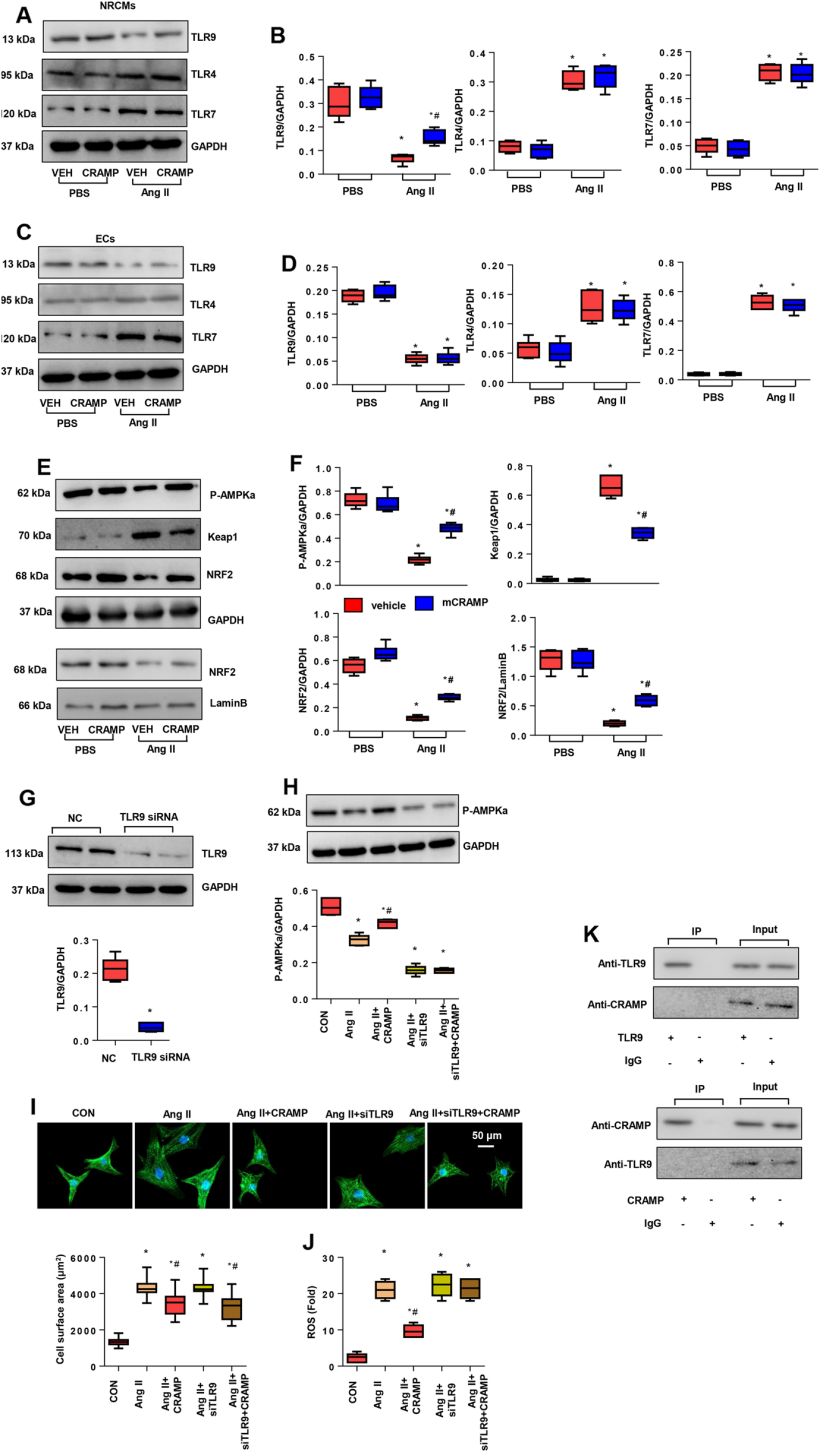
CRAMP affected TLR9/AMPKα. **a**, **b** Protein levels of TLR4, TLR9, and TLR7 in NRCMs treated with mCRAMP (0.5 mg/L) and Ang II (1 μM) (*n* = 6). **c**, **d** Protein levels of TLR4, TLR9, TLR7 in MHECs treated with mCRAMP (0.5 mg/L) and Ang II (1 μM) (*n* = 6). *P < 0.05 vs. vehicle-CON; ^#^*P* < 0.05 vs. vehicle-Ang II. **e**, **f** Protein levels of AMPKα, keap1, and NRF2 in NRCMs treated with mCRAMP (0.5 mg/L) and Ang II (1 μM) (*n* = 6). **g**–**j** NRCMs were transfected with TLR9 siRNA and treated with mCRAMP (0.5 mg/L) and Ang II (1 μM). **g** Protein levels of TLR9 in NRCMs (*n* = 6). **h** Protein levels of AMPKα (*n* = 6). **i** α-actin staining (*n* = 6) and cell surface area (*n* > 50 cells per group). **j** ROS levels (*n* = 6). **k** Co-IP experiment in NRCMs.

Studies have reported that CRAMP affects the expression pattern of toll like receptors (TLRs)^24,25^. Since TLRs are the main receptor that activates the pathology of inflammation and OS, we detected the influence of CRAMP on TLRs in NRCMs to explain the anti-OS role. We detected the membrane receptor TLR4, and cytoplasmic receptors TLR7, and TLR9 expression levels in CRAMP-treated NRCMs. As shown in Fig. 7a, TLR4 and TLR7 levels were increased, and TLR9 levels were decreased in Ang II-stimulated cardiomyocytes. CRAMP merely increased TLR9 expression levels without a change in TLR4 and TLR7 levels. We detected these TLRs in MHECs, and found that TLR4 and TLR7 levels were increased, and TLR9 was decreased in Ang II-stimulated MHECs, but CRAMP did not affect all TLRs in MHECs (Fig. 7c, d). However, the mechanism by which increased TLR9 affects OS in Ang II-stimulated NRCMs is unknown. Studies have reported that TLR9 modulates AMPKα expression in NRCMs to regulate energy metabolism^26^. We then explored the expression of AMPKα and the downstream anti-OS signaling. As shown in Fig. 7e, f, phosphorylation of AMPKα was decreased in Ang II-stimulated cardiomyocytes, but increased by CRAMP treatment. The downstream protein keap1 was increased in Ang II-stimulated cardiomyocytes but reduced by CRAMP treatment; the anti-OS protein NRF2 was reduced in Ang II-stimulated cardiomyocytes but increased by CRAMP treatment. The nuclear expression of NRF2 was also increased in CRAMP-treated cells. We used a TLR9 siRNA to silence TLR9 in NRCMs (Fig. 7g). Phosphorylation of AMPKα was decreased in the TLR9 deficient group in Ang II-stimulated cells. CRAMP treatment did not reverse this reduction in phosphorylation of AMPKα in cells with TLR9 deficiency upon Ang II stimulation (Fig. 7h). Consistently, the anti-OS effect of CRAMP was blocked by TLR9 deficiency, but the anti-hypertrophic effect of CRAMP remained (Fig. 7i, j). Co-IP assays were performed to evaluate the interaction of CRAMP and TLR9. We did not identify an interaction of CRAMP and TLR9 in NRCMs (Fig. 7k). These data suggest that CRAMP regulates OS in a failing heart by targeting the TLR9/AMPKα pathway.

### An AKT1 knockout reversed the anti-hypertrophic effect of CRAMP

**Fig. 8:**
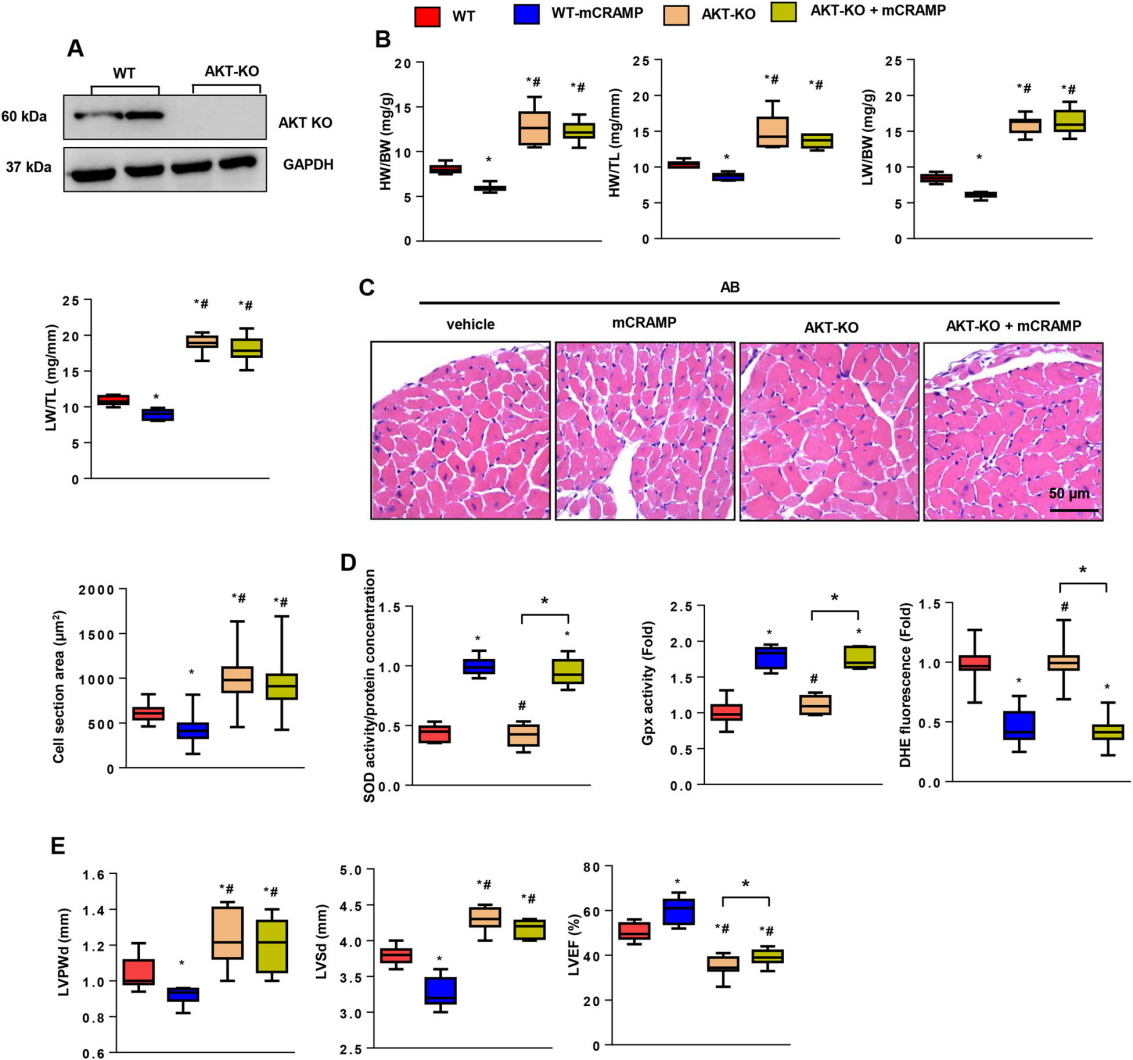
AKT1 knockout reversed the anti-hypertrophic effect of CRAMP. AKT1-KO mice were subjected to AB surgery and CRAMP treatment. **a** AKT1 protein levels in AKT1-KO mouse heart (*n* = 6). **b** HW/BW, HW/TL, LW/BW, LW/TL in mice undergoing 8 weeks of AB (*n* = 10). **c** H&E staining (*n* = 6) and cell cross-sectional area (*n* > 200 cells/group) in mice. **d** SOD, Gpx, DHE and NADPH oxidase activity in mouse heart (*n* = 6). **e** Echocardiography analysis (*n* = 8). *P < 0.05 vs. WT-AB; ^#^*P* < 0.05 vs. WT-mCRAMP.

To confirm this hypothesis in vivo, we conducted AB surgery in AKT1 knockout mice (Fig. 8a). Eight weeks after surgery, the AKT1-KO group showed a larger heart weight, lung weight, and cell cross-sectional area when compared with WT mice (Fig. 8b, c). CRAMP treatment did not relieve these negative responses (Fig. 8b, c). The OS level in AKT1-KO mice was the same as WT mice (the same levels of SOD2, Gpx, and NADPH activity), and CRAMP improved the OS levels in AKT1-KO mice (reduced NADPH activity, DHE fluorescence, and increased SOD2, and Gpx activity) (Fig. 8d). Cardiac function was worse in AKT1-KO mice with the largest LVPWd, LVSd and the lowest LVEF among these groups. The LVPWd and LVSd in CRAMP-treated AKT1-KO mice was lower than in the AKT1-KO group, but was not significantly different (*P* > 0.05); the LVEF in CRAMP-treated AKT1-KO mice was higher than AKT1-KO group (Fig. 8e). These data confirmed the hypothesis that the anti-hypertrophic effect of CRAMP was mediated by AKT1 activation in the failing heart.

### TLR9 knockout reversed the anti-OS effect of CRAMP

We then used TLR9-KO mice (Fig. S1A) to confirm our in vitro hypothesis of the anti-OS effect of CRAMP. Eight weeks after AB surgery, the TLR9-KO group revealed a larger heart weight, lung weight, and cell cross-sectional area when compared with WT mice (Fig. S1B, C). CRAMP treatment relieved these negative responses (Fig. S1B, C). The OS level in TLR9-KO mice was worse than WT mice (lower SOD2, and Gpx activity, and higher NADPH activity), and CRAMP did not affect the OS level in TLR9-KO mice (similar levels of SOD2, Gpx, DHE fluorescence and NADPH activity in CRAMP-treated and vehicle-treated TLR9-KO mice) (Fig. S1D). Cardiac function was worse in TLR9-KO mice with the largest LVPWd, LVSd and the lowest LVEF among these groups. CRAMP improved cardiac function in TLR9-KO mice with lower LVPWd, LVSd, and higher LVEF compared with vehicle-treated TLR9-KO mice (Fig. S1E). These data confirmed that the anti-OS effect of CRAMP was mediated by TLR9 activation in the failing heart.

## Discussion

Using a pressure overload-induced cardiac hypertrophy model, we provided crucial evidence that CRAMP, an antimicrobial peptide, participated in the process of pathological cardiac hypertrophy and HF. When the heart is undergoing pathological cardiac hypertrophy, CRAMP in MHECs was found to be upregulated at the beginning of the process and then decreased during the progression of HF. We discovered that when mice were treated with CRAMP, mice under pressure overload developed ameliorated cardiac hypertrophy and improved cardiac function. However, when CRAMP expression in the heart was silenced, mice developed severe cardiac hypertrophy and dysfunction. These anti-hypertrophic responses relied on the activation of the physiological cardiac hypertrophy signaling pathway IGFR/PI3K/AKT.

IGF-1 signaling regulates cardiac contraction, hypertrophy, aging, metabolism, autophagy, and apoptosis^27^. IGF-1 exerts its function by binding to membrane receptor IGF1R, which activates a 110-kDa lipid kinase PI3K subgroup Iα (p110α), leading to phosphorylation and activation of AKT1^28^. Although high levels of sustained AKT expression induced pathological growth, AKT expression protects against cardiomyocyte death and cardiac dysfunction during ischemia^29^. AKT1 has been shown to be a crucial protein that maintains cell survival and normal heart growth. Studies found that AKT1−/− mice developed greater cardiac hypertrophy in response to aortic constriction^9^. The association between CRAMP and IGF1R was first reported by Girnita A. They found that the CRAMP peptide LL-37 could bind IGF1R and its agonist via β-arrestin in malignant cells, which helped malignant cell migration and invasion^23^. In this study, we found that CRAMP exerted its anti-pathological hypertrophic effect via binding and activating IGF1R/PI3K/AKT1 signaling. In AKT1 knockout mice, the extent of pathological hypertrophy was more severe than in the WT mice, which is consistent with the findings of DeBosch B’s study. CRAMP treatment could not relieve the pathological hypertrophy in AKT1-KO mice, suggesting the central role of AKT1 in CRAMP function. Studies have reported that during the pathology of pathological cardiac hypertrophy, continuous activation of AKT signaling was observed in a pressure overload-induced mouse heart, which lead to worse cardiac function^30^. However, in our study, we did not observe continuous activation of AKT signaling, but conversely, we observed decreased activation of IGF1R/PI3K/AKT1 signaling. Further, when the activation of AKT1 was increased, pathological cardiac hypertrophy and fibrosis were ameliorated in HF mice. This inconsistency may be due to the difference in the degree of aortic constriction or different periods of HF progression.

Inflammation and OS are other features of HF. During the pathology of pathological cardiac hypertrophy, an insufficient nutrition supply leads to the injury of the mitochondria, which is the main source of ROS^31^. Increasing ROS leads to the imbalance of the redox system, causing OS^31^. Studies using various genetically modified animal models have provided more direct molecular evidence for an etiological role of ROS in cardiac remodeling and HF^31^. Previous studies have reported the anti-OS effects of CRAMP^32^. In our study, we also observed the anti-OS and anti-inflammation effects of CRAMP. However, the mechanism did not rely on AKT signaling since CRAMP exerted its anti-OS effect in the AKT1-KO mice. Thus another molecule may participate in this effect. In our experiments, we observed an increase in AMPKα/NRF2 activation after CRAMP treatment. AMPKα is an energy sensor, when the AMP/ATP ratio increases, AMPKα is activated, which activates downstream molecules that are associated with energy metabolism as well as NRF2^33^. NRF2 is transported inside the nucleus and promotes the transcription of many anti-oxidase genes^34^. In our study, we found that CRAMP increased the activation of AMPKα, consistent with Wu Ws’ study^32^, and NRF2. This may contribute to the anti-OS effect of CRAMP. However, how CRAMP interacts with AMPKα is unclear.

As an immunomodulatory peptide, CRAMP was found to affect the TLR expression pattern in immunocytes^24,25^. TLRs are a pattern recognition receptor (PRR) that triggers host inflammation. TLRs also play a crucial role in pathological cardiac hypertrophy^35^. TLR4 mediates Ang II-induced pathological cardiac hypertrophy and HF^36^. TLR7 deficiency suppresses the cardiac remodeling process induced by myocardial infarction^37^. TLR9 is essential in the pathology of the cardiac remodeling process post-myocardial infarction^38^. In our experiment, we observed an increased level of TLR4, and TLR7, and a decreased level of TLR9 expression in hypertrophic cardiomyocytes. CRAMP increased TLR9 expression without a change in levels of TLR4 and TLR7. Recent studies have found that TLR9 activates AMPK in hypoxic cardiomyocytes via an increase in the AMP/ATP ratio and by reducing substrate supply^26^. We found that the activation of AMPKa by CRAMP was mediated by increased TLR9 expression as TLR9 silencing caused suppression of AMPKa activation. Further, in our reverse experiment with the use of TLR9-KO mice, we found that the TLR9-KO showed increased pressure overload-induced pathological hypertrophy. CRAMP attenuated this pathological hypertrophy by activating IGF1R/AKT, but could not reverse the inferior redox status in the TLR9-KO failing heart. A previous study by Takafumi Oka reported that TLR9 ablation could protect against cardiac hypertrophy and HF^39^. However, Velten M later found that pre-treatment with the synthetic TLR9 ligands 1668-thioate or 1612-thioate attenuates pressure overload-induced HF^40^. CpG-ODN, classic agonists of TLR9, have also been found to attenuate pathological cardiac hypertrophy and HF by activating PI3K/AKT^41^. The findings of these two studies are consistent with our findings showing that ablation of TLR9 exerted deteriorating effects. However, the inconsistency needs further study.

In summary, our results support an important role for CRAMP in attenuating pathological cardiac hypertrophy and HF. Deficiency of CRAMP deteriorated pathological cardiac hypertrophy and HF. The anti-hypertrophic effect of CRAMP in HF relied on activation of IGF1R/PI3K/AKT1 signaling. Further, the anti-OS effect of CRAMP in HF relied on activation of TLR9/AMPKα signaling. Our study underscores the complex regulation of CRAMP in the IGF1R/PI3K/AKT1 and TLR9/AMPKα signaling loop in HF.

## Supplementary information

SUPPLEMENTAL FIGURE

Supplementary materials
