# Supplementary figures and images for: A cathelicidin-related antimicrobial peptide suppresses cardiac hypertrophy induced by pressure overload by regulating IGFR1/PI3K/AKT and TLR9/AMPKα
Source: Cell Death Dis. 2020 Feb 6;11(2):96. doi: 10.1038/s41419-020-2296-4 (PMC7005284; doi:10.1038/s41419-020-2296-4)

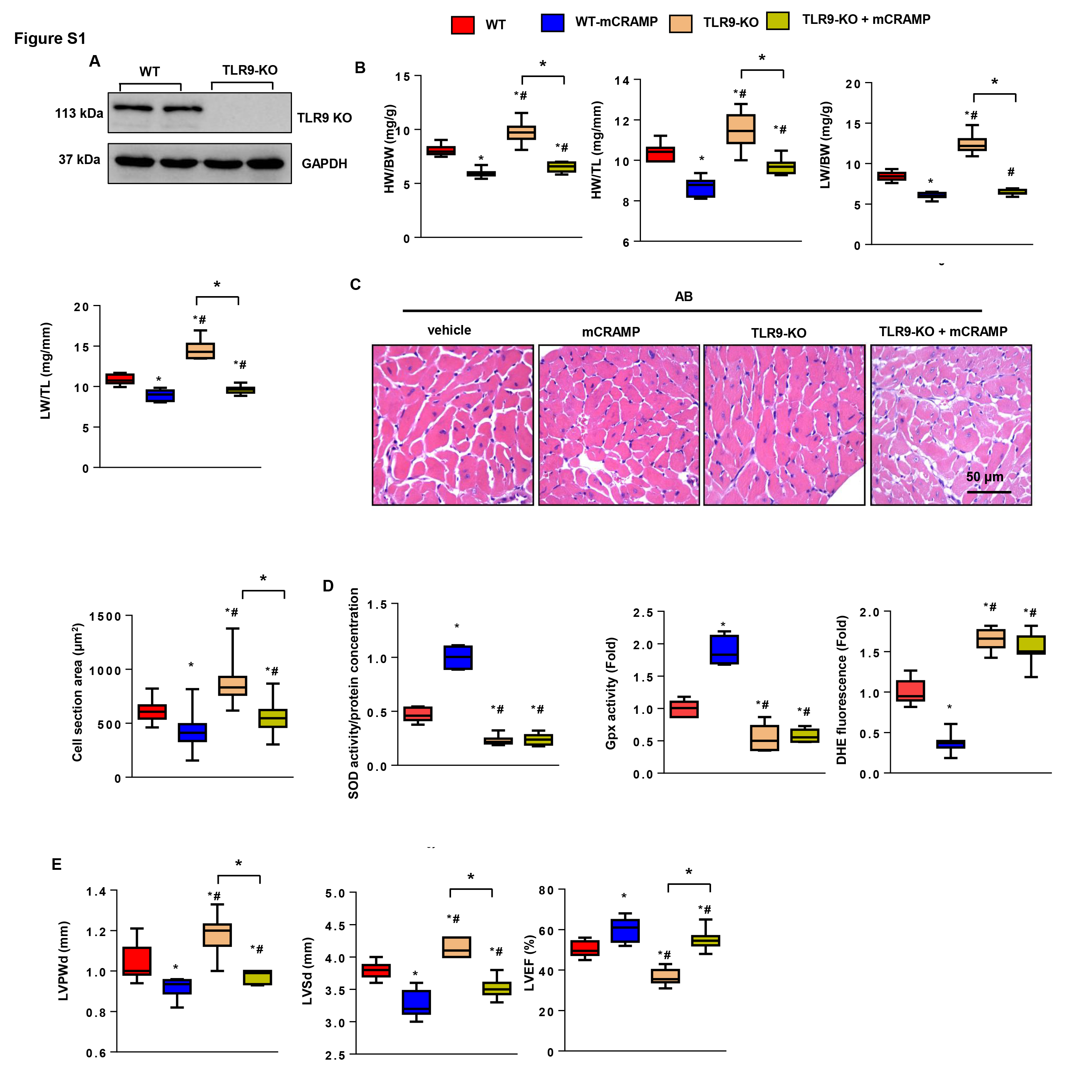

Supplement: Supplementary file 1 — SUPPLEMENTAL FIGURE [file 41419_2020_2296_MOESM1_ESM.tif]
