# Supplementary material for: A cathelicidin-related antimicrobial peptide suppresses cardiac hypertrophy induced by pressure overload by regulating IGFR1/PI3K/AKT and TLR9/AMPKα
Source: Cell Death Dis. 2020 Feb 6;11(2):96. doi: 10.1038/s41419-020-2296-4 (PMC7005284; doi:10.1038/s41419-020-2296-4)
Supplement: Supplementary file 2 — Supplementary materials [file 41419_2020_2296_MOESM2_ESM.docx]

**Figure S1. TLR9 knockout reversed the anti-OS effect of CRAMP**

TLR9-KO mice were subjected to AB surgery and CRAMP treatment. A. TLR9 protein levels in TLR9-KO mouse heart (n=6). B. HW/BW, HW/TL, LW/BW, LW/TL in mice undergoing 8 weeks of AB (n=10). C. H&E staining (n=6) and cell cross-sectional area (n > 200 cells/group) in mice. D. SOD, Gpx, DHE and NADPH oxidase activity in mouse heart (n=6). E. Echocardiography analysis (n=8). **P < 0.05* vs. WT-AB; # *P < 0.05* vs. WT-mCRAMP.
